# BRD3/4 inhibition and FLT3-ligand deprivation target pathways that are essential for the survival of human MLL-AF9^+^ leukemic cells

**DOI:** 10.1371/journal.pone.0189102

**Published:** 2017-12-14

**Authors:** Marco Carretta, Annet Z. Brouwers-Vos, Matthieu Bosman, Sarah J. Horton, Joost H. A. Martens, Edo Vellenga, Jan Jacob Schuringa

**Affiliations:** 1 Department of Experimental Hematology, Cancer Research Center Groningen (CRCG), University Medical Center Groningen, University of Groningen, Groningen, The Netherlands; 2 Department of Molecular Biology, Faculty of Science and Medicine, Radboud Institute for Molecular Life Sciences, Radboud University Nijmegen, Nijmegen, The Netherlands; European Institute of Oncology, ITALY

## Abstract

In the present work we aimed to identify targetable signaling networks in human MLL-AF9 leukemias. We show that MLL-AF9 cells critically depend on FLT3-ligand induced pathways as well as on BRD3/4 for their survival. We evaluated the *in vitro* and *in vivo* efficacy of the BRD3/4 inhibitor I-BET151 in various human MLL-AF9 (primary) models and patient samples and analyzed the transcriptome changes following treatment. To further understand the mode of action of BRD3/4 inhibition, we performed ChIP-seq experiments on the MLL-AF9 complex in THP1 cells and compared it to RNA-seq data of I-BET151 treated cells. While we could confirm a consistent and specific downregulation of key-oncogenic drivers such as MYC and BCL2, we found that the majority of I-BET151-responsive genes were not direct MLL-AF9 targets. In fact, MLL-AF9 specific targets such as the HOXA cluster, MEIS1 and other cell cycle regulators such as CDK6 were not affected by I-BET151 treatment. Furthermore, we also highlight how MLL-AF9 transformed cells are dependent on the function of non-mutated hematopoietic transcription factors and tyrosine kinases such as the FLT3-TAK1/NF-kB pathway, again impacting on BCL2 but not on the HOXA cluster. We conclude that BRD3/4 and the FLT3-TAK1/NF-kB pathways collectively control a set of targets that are critically important for the survival of human MLL-AF9 cells.

## Introduction

In the last two decades, our understanding of the molecular mechanism underlying human malignancies has greatly improved [1]. Progress in DNA-sequencing technologies has reinforced the notion that cancer is initiated and maintained by alterations in the genome and it has also become more evident that epigenetic regulators are among the most frequent aberrancies in hematopoietic malignancies [2]. Furthermore, changes in the chromatin state can also occur as a consequence of uncontrolled signal transduction activity or metabolic changes, which occur during tumorigenesis [3,4]. As a consequence, cancer cells rely on chromatin regulators to maintain a malignant phenotype [5]. These insights led to an increased interest in targeting chromatin as a therapeutic approach in cancer, with several new epigenetic therapies now evaluated in clinical trials [5,6]. One example of the latter is represented by bromodomain protein 4 (BRD3/4) inhibitors [7], which can be selectively targeted with small-molecule inhibitors like JQ1 and I-BET151 (GSK1210151A) [8,9].

BRD4 is a transcriptional and epigenetic regulator that belongs to the bromodomain and extra-terminal (BET) family of chromatin reader proteins, which also includes BRD2, BRD3, and BRDT [10]. The wild type form of BRD4 actively participates in transcription by directly phosphorylating RNA polymerase II [11] but also passively via recruitment of important transcription factors such as the RELA subunit of NF-kB [12]. Additionally, BRD4 also directly recruits P-TEFb which, through its kinase activity, promotes the elongation of RNA polymerase II [13]. BRD4 also contributes to the maintenance of chromatin structure and nucleosome clearance via its HAT activity [14]. The essential role of BRD4 in cancer was first discovered by using a negative selection RNAi screening in a mouse model of MLL-rearranged leukemia [15]. Furthermore, it has been shown that the small molecule inhibitor of the BET family I-BET151 (GSK1210151A) is efficient against human and murine MLL-fusion leukemic cell lines, through the induction of early cell cycle arrest and apoptosis [8,9]. It was suggested that the mode of action of this inhibitor is in part due to the inhibition of key genes through the displacement of BRD3/4, PAFc and SEC components from the chromatin.

Chromosomal translocations involving the MLL gene define a unique group of leukemias, that can give rise to acute myeloid leukemia (AML), acute lymphoblastic leukemia (ALL) or biphenotypic leukemia (BAL) and they are generally associated with poor prognosis [16]. MLL fusions are transcriptional regulators that take control of targets normally controlled by MLL. Within wild type MLL the SET domain confers H3K4 methyltransferase activity, allowing transcription initiation by Polymerase II [17]. When the MLL gene is fused with one of its partners, such AF9, the SET domain is lost together with its catalytic activity. However, MLL fusion proteins gain the ability to methylate H3K79, which results in aberrant gene expression of homeobox genes such HOXA9 and MEIS1. Furthermore, the H3K79 methyltransferase DOT1L and the MLL-interacting protein Menin have emerged as important mediators of MLL fusion-driven leukemic transformation [18].

It is remarkable to note that, although its inhibition causes potent anti-proliferative effects in various leukemic sub-types, BRD4 is generally not mutated in cancer and normal hematopoietic cells show no sensitivity to this inhibitor. Experimental evidence so far supports the notion that the anti-proliferative effects observed in MLL-fusion driven leukemia upon inhibition of BRD4 was, at least in part, due to the downmodulation of MYC, BCL2 and CDK6 [8,19] but it has remained unclear whether BRD4 is directly recruiting the MLL-AF9 protein to chromatin or whether BRD4 inhibition would act on independent molecular pathways. In the present work, we tested the I-BET151 efficacy in an MLL-AF9 transduced cord blood model *in vitro* and *in vivo*, and also on primary patient samples. By performing genome-wide transcriptome and ChIP-seq experiments we found that the majority of I-BET151-responsive genes were not specific MLL-AF9 targets. In fact, only a limited number of direct MLL-AF9 target genes including key-oncogenic drivers such MYC and BCL2 were sensitive to I-BET151, while the expression of other MLL-AF9 specific targets such as the HOXA cluster, CDK6 and MEIS1 was unaffected. MLL-AF9 cells are also critically dependent on the activity of FLT3 and TAK1/NF-kB pathways to maintain proliferation and survival and, like BRD4, these pathways control MYC and BCL2 in an MLL-AF9-independent manner.

## Material and methods

### Primary cell isolations

Neonatal CB samples were obtained after informed consent from healthy full-term pregnancies from the obstetrics departments of the University Medical Center in Groningen (UMCG) and Martini Hospital Groningen. BM mononuclear cells from untreated patients were studied after informed consent and protocol approval by the Medical Ethical Committee of the UMCG, in accordance with the Declaration of Helsinki. All studies were approved by the Intitutional Review Board. After ficoll separation of mononuclear cells, CD34^+^ cells were enriched using a magnetically activated cell-sorting CD34 progenitor kit or automatically by using auto Macs (Miltenyi Biotech) as described previously [20,21] and cryopreserved until further use.

### Lentiviral transductions

CB CD34^+^ cells were pre-stimulated and transduced as described previously [20–22]. One round of transduction was performed and cells were harvested at day 2 after transduction. For MLL-AF9 transformation of CB CD34^+^ cells UMG LV6 MLL-AF9 lentiviral vectors were used [23]. THP1 cells were transduced with a lentiviral vector expressing human BCL2 in which the EGFP cDNA was exchanged for mBlueberry2 cDNA which was a kind gift of Robert Campbell, University of Alberta, Canada [24].

### Cell culture

Leukemic cell lines MOLM13, THP1 and K562 (all obtained from the American Type Culture Collection, ATCC) were cultured in RPMI 1640 (Lonza, Leusden, The Netherlands) supplemented with 10% fetal bovine serum and 1% penicillin and streptomycin. CB transduced MLL-AF9 cells in the liquid and MS5 myeloid co-culture experiments were grown in Gartner’s medium consisting of αMEM (Fisher Scientific Europe, Emergo, The Netherlands) supplemented with 12.5% heat-inactivated fetal calf serum (Lonza, Leusden, The Netherlands), 12.5% heat-inactivated horse serum (Invitrogen, Breda, The Netherlands), 1% penicillin and streptomycin, 2mM glutamine (all from PAA Laboratories), 57.2 μM β-mercaptoethanol (Merck Sharp & Dohme BV, Haarlem, The Netherlands) and 1 mM hydrocortisone (Sigma-Aldrich Chemie B.V., Zwijndrecht, The Netherlands). CB MLL-AF9 myeloid restricted cultures were supplemented with 20 ng/ml IL-3, SCF and FLT3-L (R&D Systems). Lymphoid permissive co-cultures contained the same components as the myeloid cultures with the exception of hydrocortisone and horse serum but with the presence of 50 μg/ml ascorbic acid (Sigma). In lymphoid CB MLL-AF9 co-cultures IL-3 was replaced with 10 ng/ml IL-7 (R&D Systems). AML cells in myeloid restricted cultures were supplemented with 20 ng/ml IL-3, SCF, FLT3-L, granulocyte colony-stimulating factor (Rhone-Poulenc Rorer, Amstelveen, The Netherlands), and thrombopoietin (Kirin, Tokyo, Japan) as previously described [25–27]. All the cultures were kept at 37°C and 5% CO2. During demi-depopulation of cultures, it was made sure that stromal cells remained attached and that only hematopoietic/leukemic cells were included in the analyses (as also validated by flow cytometry for EGFP in case of transduced cells or by staining with huCD45 when appropriate). For all experiments (except for data shown in Fig 4D) week 6–10 immortalized CB MLL-AF9 cultures were used, or where indicated cell lines or primary patient samples, and the molecular consequence upon inhibition of BRD3/4, NF-kB or TAK1, or upon deprivation of FLT3-L were analysed within 1–6 days as indicated in the respective figures. I-BET151 (GSK GSK1210151A) was kindly provided by Nicholas Smithers (GSK R&D, UK), the TAK1 inhibitor 5z-7-oxozeanol was obtained from Sigma and the IKK VII inhibitor was obtained from Abcam (ab216471, Abcam).

### FACS analysis and cell sorting

All FACS analyses were performed on a FACS Calibur (Becton Dickinson) or LSR-II (Becton Dickinson) and data were analysed using Flow Jo (Tree Star, Inc.). Cell sorting was performed on a MoFlo-Astrios (Beckman Coulter). All antibodies were obtained from IQ products. The following clones were used: Annexin V R-PE (IQP-120R), Annexin V APC (IQP-120A) and Annexin V FITC (IQP-120F). Cells were washed in calcium buffer (10 mM HEPES/NaOH, pH 7.4, 140 mM NaCl) and incubated with antibodies at 4°C for 30 min.

### *In vivo* treatment study

The ectopic bone model was established as described previously [28,29]. Briefly, four hybrid scaffolds consisting of three 2–3 mm biphasic calcium phosphate particles loaded with human MSCs were implanted subcutaneously into 6 to 8 weeks old female NOD.Cγ-Prkdcscid Il2rγtm1Wjl/SzJ (NSG) mice. Six to eight weeks after scaffold implantation, 0.5x10^6^ cells were directly injected into 3 out of the 4 scaffolds. Human CD45 engraftment was analysed by timely sub-mandibular bleeding procedures. Control mice were injected with normal saline containing 1% (v/v) DMSO. Treated mice were inject with 500μM and 100 μM I-BET151 dissolved in normal saline containing 1% DMSO (v/v), 10% Kleptose HPB (w/v) and 2% HCl. Tumor sizes were measured by a caliper and tumor volumes were calculated by the modified ellipsoidal formula: V = 0,5x(LxW^2^) where V is the volume, L the greatest longitudinal diameter (length) and W the greatest transverse diameter (width). All animal studies were approved by our Animal Ethical Committee at the UMCG. Animals were monitored twice daily in order to identify any suffering or distress. Humane endpoints used were: tumor volume should not exceed 2 cm^3^ (conform the Code of Practice at the Animal Ethical Committee UMCG) or chimerism in the peripheral blood of leukemic blasts should not exceed 50%. Mice were euthanized by cervical dislocation under isoflurane treatment. We did not observe mortality outside the planned euthanasia or humane endpoints.

### qPCR

Total RNA was isolated using the RNeasy micro kit from QIAGEN (Venlo, the Netherlands) according to the manufacturer’s recommendations. RNA (250 ng) was reverse transcribed with MMLV reverse transcriptase (Biorad). cDNA was realtime amplified in iQ SYBR Green supermix (Biorad) with the MYIQ thermocycler (Biorad). Primer sequences are available on request. RPL27, HPRT and B2M were used as housekeeping genes.

### Transcriptome analysis

Total RNA from immortalized CB MLL-AF9 cells co-cultured with MS5 cells (week 6–10) was isolated using the RNeasy micro kit from Qiagen (Venlo, The Netherlands) according to the manufacturer’s recommendations. RNA quality was examined using the Experion RNA StSense kit (BioRad). Genome-wide expression analysis was performed on Illumina (Illumina, Inc., San Diego, CA) HumanHT-12 v4 Expression BeadChip (47k probesets). Typically, 200ng mRNA for amplification with Illumina TotalPrep 96RNA Amplification Kit (Ambion) and 750ng of cRNA was used in labeling reactions and hybridization with the arrays according to the manufacturer’s instructions. Data were analyzed using the GenomeStudio V2011.1 (Illumina, Inc.) and Genespring 12.6.1 (Agilent, Amstelveen, The Netherlands). Clustering analyses were performed using Genespring software. For RNA sequencing, total RNA from THP1 cells was isolated using the RNeasy micro kit from Qiagen (Venlo, The Netherlands) according to the manufacturer’s recommendations. An initial quality check and RNA quantification of the samples was performed by capillary electrophoresis using the LabChip GX (Perkin Elmer). With 50ng mRNA sequence libraries were generated using the Lexogen Quantseq 3’ prep kit (Lexogen GmbH) according to the manufacturer’s recommendations. The obtained cDNA fragment libraries were sequenced on an Illumina NextSeq500 using default parameters (single read). All the bioinformatics were performed on the Strand Avadis NGS (v3.0) software (Strand Life Sciences Pvt.Ltd). Raw sequence quality was checked for GC content, base quality and composition using FASTQC and StrandNGS. Quality trimmed reads were aligned to build a Human HG19 (UCSC) transcriptome. Ensembl genes and transcripts (2014.01.02) were used as gene annotation database. Quantified reads were normalization using the DESeq package. Reads with failed vendor QC, reads with average quality less than 24, reads with mapping quality below 50 and reads with length less than 20 were all filtered out.

### Chromatin immuno precipitation

ChIP was essentially performed as described previously [30]. ChIP reactions were performed using the following antibodies: anti-H3K27Ac (ab4729, Abcam), anti-H3K79me2 (ab3594, Abcam), anti-BRD4 (Ab128874, Abcam). ChIP efficiencies were determined by qPCR (primers available on request). MLL, AF9, H3K79me2, H3K4me3, H3K27ac and H3K27me3 ChIP-seq data in THP1 cells used in this current manuscript were published previously [31].

### Statistics

Unpaired two-sided Student’s test was used to calculate statistical differences. A p value of <0.05 was considered statistically significant (*p<0.05, **p<0.01, ***p<0.001).

## Results

### I-BET151 inhibits MLL-fusion leukemic cell lines, cord blood models and patient derived cells *in vitro* and *in vivo*

**Fig 1:**
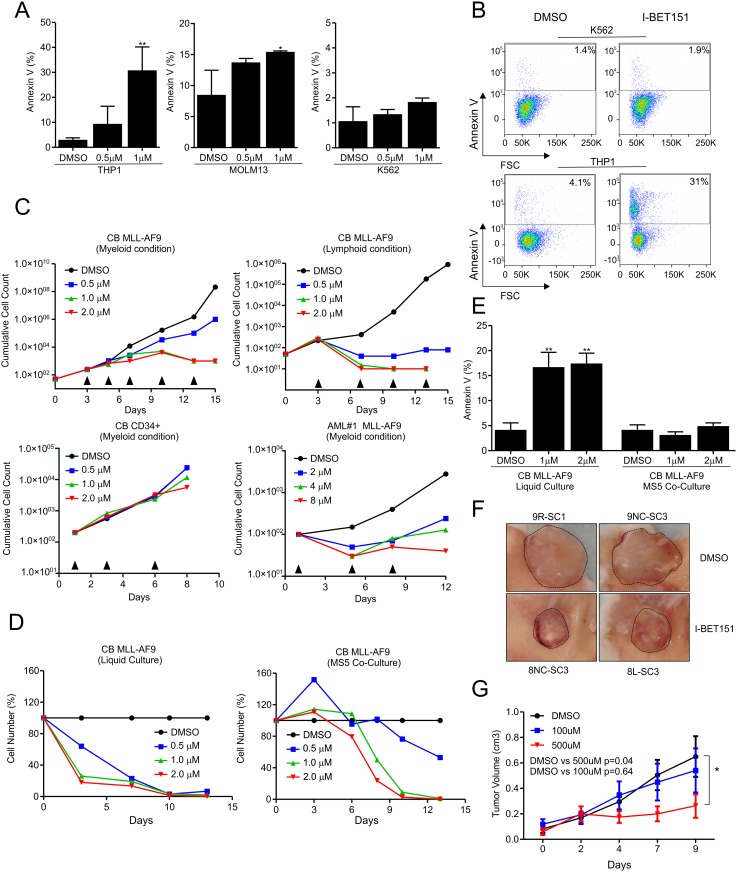
I-BET151 inhibits MLL-fusion leukemic cell lines, patient-derived cells and cord blood models *in vitro* and *in vivo*. **A**) Percentage of Annexin V positive cells after 72 hours incubation with DMSO or I-BET151 in different concentrations (technical triplicate ± s.d of 3 independent experiments) for different human leukemia cell lines. **B**) FACS analysis of Annexin V positive cells after 72 h incubation with DMSO or 1 μM I-BET151. **C**) Growth curves for immortalized CB MLL-AF9 MS5 co-cultures (myeloid and lymphoid conditions), normal CB CD34^+^ cells and patient derived CB MLL-AF9 (AML#1) upon I-BET151 treatment. Arrows indicate treatment. For CB transductions, demi-depopulated cells were analysed by flow and all cells were GFP^+^ and cell counts reflect MLL-AF9^+^ CB cells. **D**) Growth curves for immortalized CB MLL-AF9 myeloid cells in MS5 co-culture and liquid condition. Data were normalized to 100% relative to the response of DMSO treated cells. I-BET151 was added at every indicated time point. **E**) Percentage of Annexin V positive cells after 72 h incubation with DMSO or I-BET151 in different concentrations (technical triplicate ± s.d of 3 independent experiments) for CB transduced MLL-AF9 in liquid or MS5 co-culture setting. **F**) CB MLL-AF9 cells were transplanted into human Scaffold mice after which mice were treated with I-BET151 and pictures depict tumors in control groups and 500uM I-BET151 treated groups. **G**) Experiment as in H, and tumor volumes were measured by caliper during the course of the experiment.

First, we verified the therapeutic potential and specificity of the BRD3/4 inhibitor I-BET151 (GSK1210151A) for leukemic cell lines harbouring MLL-fusion oncogenes. Annexin V levels were measured after 72 hours incubation with dimethyl sulfoxide (DMSO) or different dosages of I-BET151. Significantly higher and dose-dependent levels of Annexin V positive cells were detected for MOLM13 and THP1 treated cell lines, while the K562 cell line (harbouring the BCR-ABL translocation) was not affected by the treatment (Fig 1A and 1B). Next, immortalized CB-transduced MLL-AF9 cells were grown for two weeks under myeloid MS5 co-culture conditions in the absence or presence of I-BET151 and reduced expansion was observed in a dose-dependent manner, while normal CB CD34^+^ cells were not sensitive (Fig 1C and S1A Fig). When CB transduced MLL-AF9 cells were transformed along the B-ALL lymphoid lineage [22] cells were also sensitive to I-BET151 (Fig 1C and S1B Fig). In addition to CB MLL-AF9 transduced models, I-BET151 also strongly impaired proliferation of primary MLL-AF9 patient cells (Fig 1C).

Intriguingly, when we evaluated the response to I-BET151 under liquid culture conditions, we observed a much faster and stronger response to I-BET when compared to MS5 cocultures (Fig 1D). In line with these observations, Annexin V levels were already increased within 72 hrs of I-BET151 treatment in CB MLL-AF9 cells grown under liquid culture conditions but not when grown under MS5 stromal coculture conditions (Fig 1E). These data suggest that the presence of a bone marrow microenvironment provides protective signals reducing I-BET151 sensitivity.

Lastly, in order to evaluate the therapeutic potential of I-BET151 *in vivo*, we took advantage of a humanized bone marrow xenograft model (huBM-sc) of secondary MLL-AF9-driven B-ALL that we have previously published [29]. 5 × 10^5^ cells harvested from a primary MLL-AF9-driven B-ALL generated in huBM-sc mice were injected into three huBM scaffolds of secondary recipients. Five weeks after injection of the cells, we performed daily intra-scaffold (i.sc.) injections of the inhibitor for a total of 9 days. One control group (n = 3) was injected with a solution of phosphate-buffered saline and 1% DMSO, one group (n = 3) with I-BET151 100 μM and another group (n = 3) with I-BET151 500 μM. The tumor volume of each scaffold was measured every 2 days, allowing us to monitor the progression of the disease in time. All mice were sacrificed at the same time 44 days after injection. Mice treated with 500 μm I-BET151 displayed a significant reduction in tumor volume (p = 0.04) (Fig 1F and 1G). No significant differences were found in spleen weight or blood and BM chimerism. The effects on overall survival could not be assessed since mice needed to be sacrificed due to tumor size in order to adhere to the predefined human endpoints of the experiment. Since mice needed to be sacrificed Together, these data indicate that I-BET151 treatment significantly delays lymphoid leukemia progression *in vivo* but is not sufficient for a complete eradication of the leukemic cells, at least not in this experimental setting.

### Downmodulation of BCL2 and C-MYC but not HOXA genes upon I-BET151 treatment

**Fig 2:**
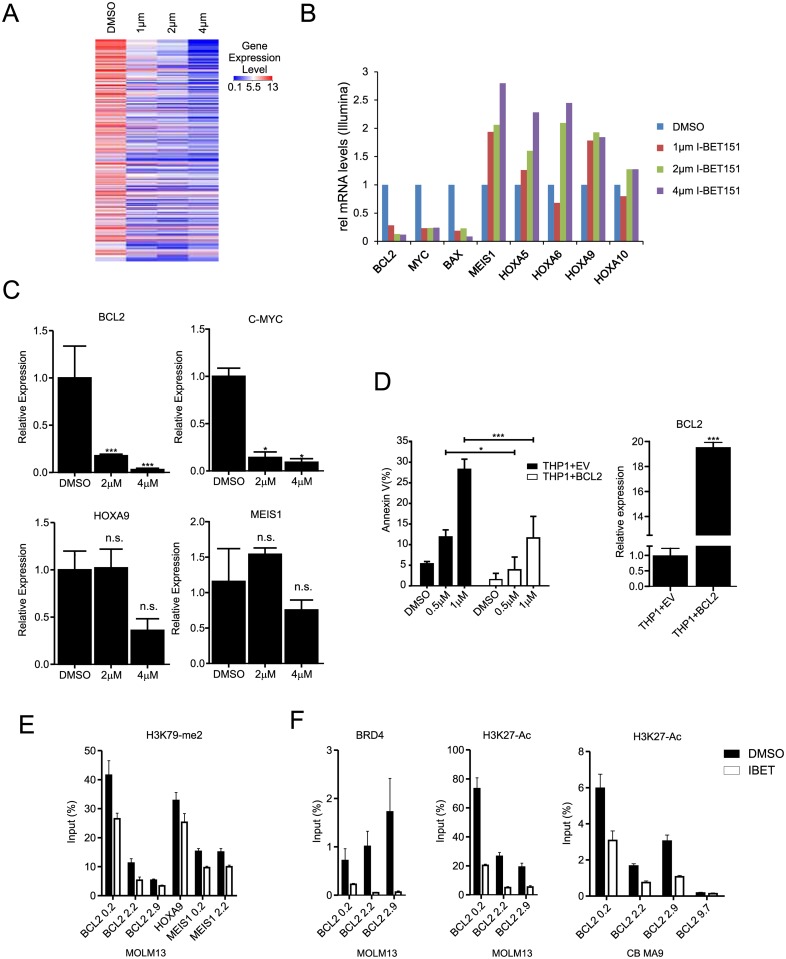
Downmodulation of BCL2 and C-MYC upon I-BET151 treatment. **A**) Heatmap of top 306 downregulated genes (at least 2-fold, in a dose dependent manner) in immortalized CB MLL-AF9 cells grown in MS5 stroma following treatment with I-BET151. B) Examples of the Illumina gene array data indicating a strong downregulation of BCL2, MYC and BAX, but not HOXA genes upon treatment with I-BET151. C) Validation of gene array data by Q-PCR of a selected subset of genes (normalized to RPL27 expression). **D**) Overexpression of BCL2 in THP1 cells reduces apoptosis as determined by the number of Annexin V positive cells levels after 24h incubation with 2 μM I-BET151. The level of BCL2 overexpression in transduced cells is also shown. **E**) MOLM13 cells were treated with 2 μM I-BET151 for 48 hrs after which ChIP-PCR analysis was performed using primers for BCL2, HOXA9 and MEIS1 loci using anti-H3K79-me2 antibodies. The distance from the transcription start site (in kb) is indicated. Bar graphs are represented as the mean enrichment relative to input and error bars reflect standard deviation of results derived from technical triplicate experiments. **F**) ChIP-PCR experiments as in E, but now antibodies against BRD4 or H3K27-Ac were used.

In order to gain more insight in the transcriptional pathways regulated by I-BET151, genome-wide transcriptome analysis was performed in myeloid immortalized CB MLL-AF9 cells after treatment with DMSO or I-BET151 in an MS5 co-culture setting. Since cells cultured on the MS5 stromal feeder displayed a slower response to BRD3/4 inhibition, RNA was isolated after 6 days, following two rounds of treatment. Cells were treated with 1 μM, 2 μM, 4 μM or with DMSO controls. Thus we identified 306 genes that were commonly downregulated at least 2 fold compared to DMSO treated cells and that responded to the inhibitor in a dose-dependent manner (Fig 2A). In accordance to previously published observations, we found a consistent downmodulation of transcription factors such cMYC and anti-apoptotic genes like BAX and BCL2 (Fig 2B). Surprisingly, well-known MLL-AF9 targets such as the HOXA gene cluster and MEIS1 were not significantly affected by the treatment, as we also confirmed by independent Q-RT-PCR experiments (Fig 2B and 2C).

BCL2 is a crucial anti-apoptotic gene and its role in MLL-AF9 driven leukemia has already been investigated [32]. To assess whether we could rescue the apoptotic phenotype upon I-BET151 treatment, we overexpressed BCL2 in THP1 cells. After 72 hours incubation with different I-BET151 dosages, a significant reduction of Annexin V cells was observed in the THP1 cells overexpressing BCL2 (Fig 2D).

Since the direct MLL-AF9 target genes HOXA9 and MEIS1 were not downregulated upon I-BET151 treatment we investigated whether BRD3/4 inhibition would directly affect the MLL-AF9 oncoprotein complex and the activity of the methyl transferase DOT1L by performing ChIP-qPCRs for H3K79me2. While we observed a slight reduction in H3K79 dimethylation on all the tested loci upon treatment with 2 μM I-BET151, the loss of H3K79me2 marks was not very strong (Fig 2E), suggesting that indeed MLL-AF9 itself is not particularly affected by I-BET151. In order to confirm that I-BET151 was targeting BRD3/4 we performed ChIP-PCRs for BRD4 and H3K27-ac on the BCL2 locus in MOLM13 and CB MLL-AF9 cells and as shown in Fig 2F a strong reduction in both BRD4 binding as well as acetylation of H3K27 was observed, in line with the strong reduction in BCL2 expression.

### BRD3/4 inhibition downregulates MLL-AF9-dependent and independent target genes

**Fig 3:**
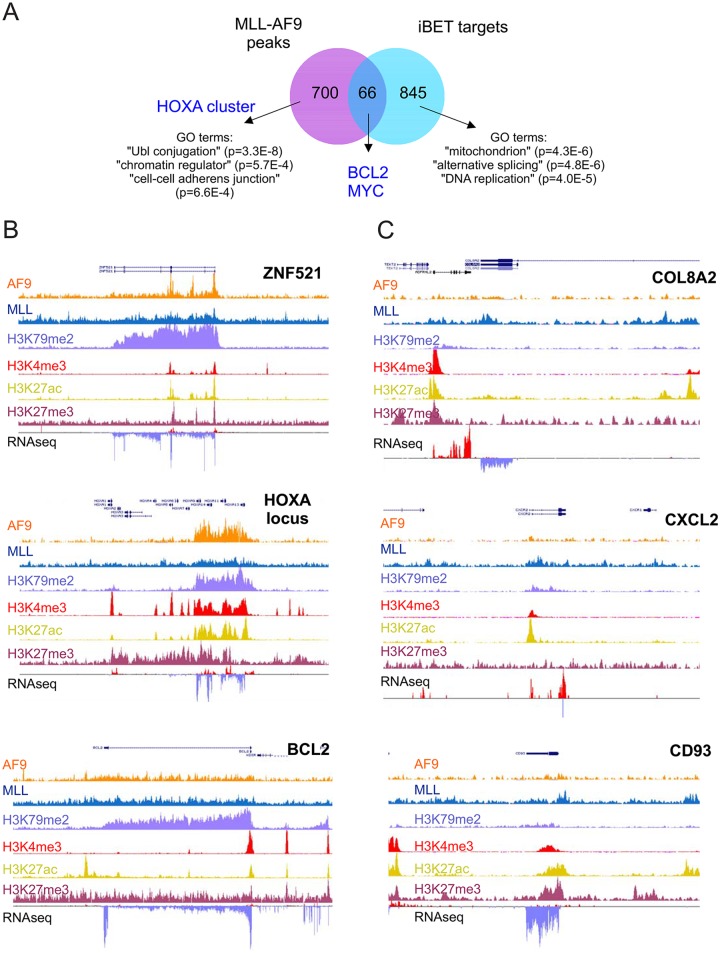
BRD3/4 inhibition downregulates MLL-AF9-dependent and independent target genes. **A**) Venn diagram for MLL-AF9 specific binding sites in THP1 cells [31] versus 3-fold downregulated genes upon 2 μM I-BET151 treatment in THP1 cells in a dose-dependent manner. **B**) ChIP-seq occupancy profiles of AF9, MLL, H3K79me2, H3K4me, H3K27Ac, H3K27me3 at the various loci. Examples of specific MLL-AF9 gene targets are shown. RNAseq in THP1 cells was also performed and tracks are included as well. **C**) As in B but now examples of non-specific MLL-AF9 gene targets are shown.

While BRD3/4 inhibition significantly impaired proliferation and survival of MLL-AF9 driven leukemia in both *in vitro* and *in vivo* studies, the involved targets did not seem to completely overlap with the MLL-AF9-induced oncogenic program. In order to gain more insights in the mechanism of action of the I-BET151 inhibitor, we compared MLL-AF9-occupied loci in THP1 cells using ChIPseq data [31] with RNA-seq data upon 24h of I-BET151 treatment. A total of 766 MLL-AF9 occupied loci were identified using an association rule of ± 10 kb from the transcription start site (Fig 3 and S1 Table) [31]. As shown in Fig 3A, the overlap of these loci with the genes down regulated in THP1 cells upon 24h I-BET151 treatment was very limited. While we identified 911 downregulated genes in THP1 upon 2 μM I-BET151 treatment in a dose-dependent manner, only 66 of these were also bound by MLL-AF9 indicating that I-BET151 targets only few MLL-AF9 direct target genes. Gene ontology (GO) annotation for the non-overlapping downmodulated genes upon I-BET151 treatment on THP1 were significantly enriched for ‘Transcription’ and ‘DNA replication’ involving genes such as MYB, FOXM1 and various members of the E2F family of transcription factors. As expected, instead, the MLL-AF9 specific and non-overlapping genes were enriched for GOs such ‘chromatin regulator’ and including genes such the HOXA cluster, MEIS1, BM1, CBX8 and other known MLL-AF9 specific targets. Examples of MLL-AF9 specific and non-specific binding sites are displayed in Fig 3B, together with H3K79me2, H3K4me3, H3K27Ac and H3K27me3 chromatin modifications tracks.

Interestingly, we did observe MLL-AF9 binding and H3K79-me2 deposition on BCL2 and MYC, genes that were also downregulated upon I-BET151 treatment. Clearly, MLL-AF9 binding might contribute to expression of these loci, but the sensitivity to I-BET151 is particularly mediated via BRD3/4 independent of MLL-AF9.

Interestingly, among the upregulated genes upon I-BET151 treatment we have found genes that negatively control cell cycle such as CDKN1A and CDKN1B and again a rather limited overlap with MLL-AF9 occupied loci was observed (S2A Fig and S1 Table). The upregulated genes enriched for GO terms such as R-SMAD binding, G2/M transition of mitotic cell cycle and negative regulation of cell proliferation (S2A Fig) and it is quite possible that these upregulated cell cycle inhibitors further contribute to the negative phenotype imposed on MLL-AF9 cells by BRD3/4 inhibition.

### MLL-AF9 cells critically depend on FLT3

**Fig 4:**
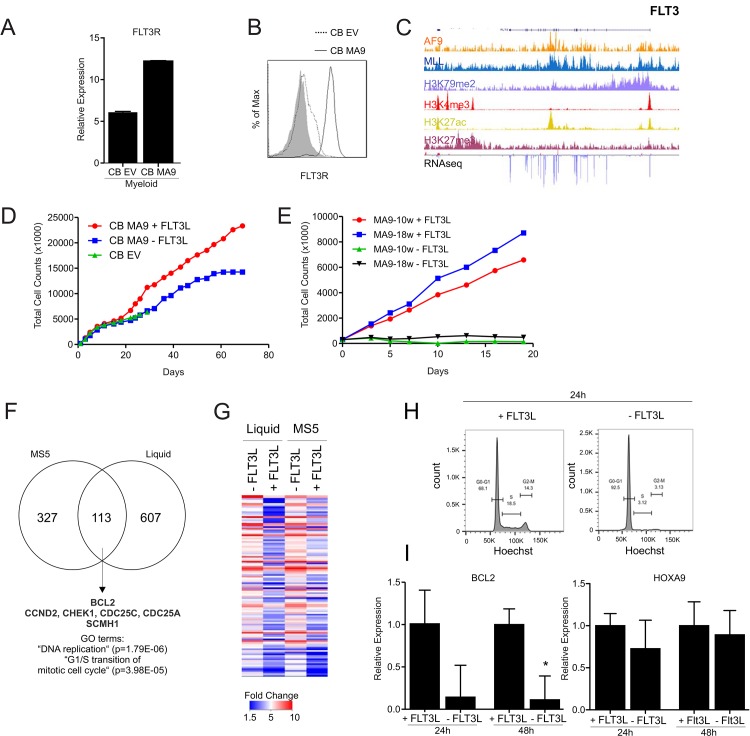
BCL2 downmodulation upon FLT3-L deprivation. **A**) mRNA expression of the FLT3 receptor in CB transduced MLL-AF9 cells (myeloid and lymphoid) versus empty vector control. **B**) FACS analysis of the FLT3 receptor in myeloid CB transduced MLL-AF9 cells versus empty vector control. **C**) ChIP-seq occupancy profiles of AF9, MLL, H3K79me2, H3K4me, H3K27Ac, H3K27me3 at FLT3 locus in THP1 cells. RNAseq tracks are shown as well. **D**) Total cumulative cell count for MS5 co-culture of freshly transduced CB MLL-AF9 cells under myeloid restrictive condition, in the presence or absence of the FLT3 ligand. **E**) Total cumulative cell count for week 10 or week 18 transformed CB MLL-AF9 cells on MS5 stroma that were deprived of FLT3-L or not for a period of 2.5 weeks. **F**) Venn diagram representing genes down-regulated at least 2-fold in immortalized CB MLL-AF9 cells in the presence or absence of FLT3L after 48 hours in liquid cultures or in MS5 cocultures. **G**) Heatmap displaying the 113 commonly down-regulated genes in liquid and MS5 co-culture setting. **H**) Cell cycle analysis by FACS of immortalized CB MLL-AF9 cells in the presence or absence of the FLT3-L after 24h in liquid culture conditions. **I**) Q-RT-PCRs were performed to determine gene expression levels of BCL2 and HOXA9 after 24 and 48 hours of FLT3L deprivation.

In our previous studies aimed at better understanding molecular mechanisms sustaining MLL-AF9-induced transformation we identified FLT3 as a strongly upregulated MLL-AF9 target [22]. Here, we further confirmed this upregulation at the RNA and protein level (Fig 4A and 4B). We also observed direct MLL-AF9 binding and H3K79-me2, H3K4-me3 and H3K27-Ac deposition at the FLT3 locus (Fig 4C). To functionally study the role of the FLT3 receptor in CB MLL-AF9 cells during the initiation phase of transformation, freshly isolated CD34^+^ CB cells were transduced with the MLL-AF9 fusion gene and cultured in the presence or absence of the FLT3 ligand (FLT3-L) on MS5 stroma under myeloid-restricted conditions. After two to three weeks of FLT3-L-deprivation a reduction in proliferation of CB MLLAF9 cells was observed, which became progressively more evident after the third week of culture (Fig 4D). These data indicate that FLT3-L is required for the initiation of transformation induced by MLL-AF9, which usually starts to occur around week 3 [22]. After 4 weeks in culture, no differences in Annexin V levels where detected between cells supplied with or without the FLT3-L, indicating that cells are not entering apoptotic program (data not shown).

We also questioned whether FLT3-L was required for the maintenance of MLL-AF9 transformed cells. To evaluate this, we used week 10 or week 18 immortalized CB MLL-AF9 cultures, and as shown in Fig 4E, removal of FLT3-L also had detrimental effects on immortalized cultures.

Next, we sought to analyse the changes in gene expression upon deprivation of FLT3-L under leukemia maintenance conditions. RNA from immortalized CB MLL-AF9 cells grown in MS5 co-culture or liquid culture conditions were isolated after 48 hours. Interestingly, down-regulation of genes was stronger in liquid culture conditions compared to the MS5 co-cultures, possibly due to the fact that stromal cells are producing low levels of FLT3-L. 113 genes were commonly downregulated after 48 hours in both liquid and co-culture settings which were significantly enriched for GO terms such as Cell Cycle, Cell Division and G1/S transition of mitotic cell cycle (Fig 4F and S1 Table). Indeed, genes involved in cell cycle regulation such CND2, CHEK1, CDC25C and CDC25A were found to be downregulated upon FLT3-L deprivation, and a marked reduction of ~25% in cells in S/G2/M phase was found in cells deprived of the FLT3-L after 24 hours (Fig 4H).

As observed for the I-BET151 treated cells, a component of the PcG complex SCMH1 and the anti-apoptotic BCL2 were also found to be consistently downregulated (Fig 4I), suggesting that FLT3-L and BRD3/4 pathways converge at a set of target genes including BCL2. Moreover, and once again as observed for the I-BET151 treated cells, none of the HOXA cluster of genes was affected by the ligand deprivation as confirmed also by qPCR for HOXA9 (Fig 4I). Taken together, these data reinforce the notion that although certain expression programs are directly regulated by the MLL-AF9 oncoprotein complex, leukemic transformation still critically depends on additional pathways.

### Inhibition of the TAK1-NFkB axis phenocopies FLT3-L deprivation

**Fig 5:**
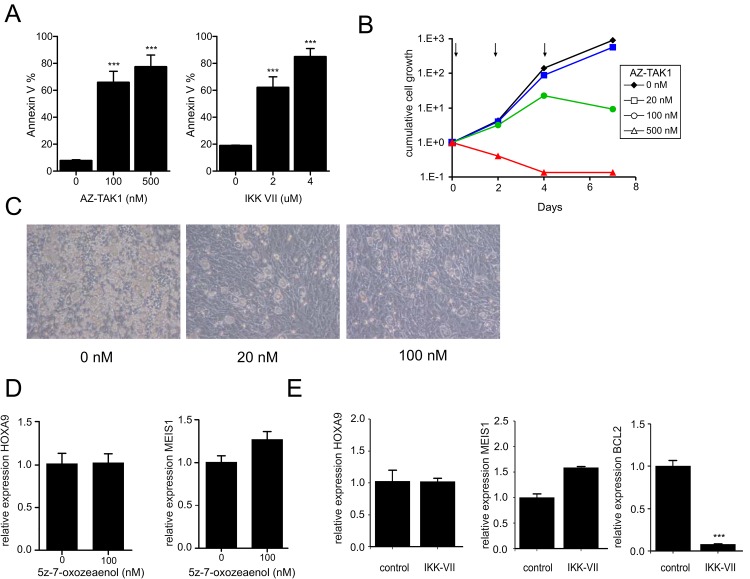
Inhibition of the TAK1-NFkB axis phenocopies FLT3-L deprivation. **A**) Percentage of Annexin V-positive CB MLL-AF9 cells after 24 hours incubation with DMSO or AZ-TAK1 inhibitor or IKK VII in different concentrations (technical triplicate ± s.d of 3 independent experiments). **B**) Growth curves for immortalized CB MLL-AF9 MS5 co-cultures treated with different concentrations of AZ-TAK1 inhibitor (arrows indicate treatments; a representative of three independent experiments is shown). **C**) Microscope images of immortalized CB MLLAF9 MS5 co-cultures. Cobblestone formation was reduced upon TAK1 inhibitor treatment. **D**) qPCR on CB MLL-AF9 cells after 24 hours treatment with 5z-7-oxoezeanol or IKK VII inhibitor (**E**).

Next, we aimed to further identify the pathways downstream of the FLT3 receptor that are critically important for the survival and proliferation of MLL-AF9 cells. Recently, it was shown that MLL oncoproteins, such as MLL-AF10, are dependent on IKK/NF-kB activity for their survival [33]. Furthermore, there is experimental evidence showing that FLT3 activation either by its ligand or by oncogenic mutations results in activation of NF-kB to support cell survival [33–37]. We analysed whether inhibition of IKK/NF-kB or one of its upstream activators TAK1 would phenocopy the effects observed upon FLT3L deprivation in MLL-transformed cells. Treatment with either the TAK1 inhibitor AZ-TAK1 or the IKK VII inhibitor for 24 hrs resulted in strongly enhanced apoptosis of immortalized CB MLL-AF9 transformed cells (Fig 5A). Cell proliferation was also severely impaired on MS5 stromal cocultures by AZ-TAK1 (Fig 5B) or the IKK VII inhibitor (data not shown). Importantly, also a concentration-dependent reduction in cobblestone formation and maintenance was seen upon treatment with AZ-TAK1 (Fig 5C) implying that the primitive transformed leukemic cells were also targeted by the TAK1 inhibitor. To study whether the TAK1/NF-kB axis would act independently of MLL-AF9 we determined the expression of HOXA9 and MEIS, and we observed that the expression of both transcripts was not affected upon TAK1 or IKK VII inhibition (Fig 5D and 5E). In contrast, these inhibitors resulted in a marked reduction of BCL2 expression (Fig 5E and data not shown) indicating that, similarly to what observed upon FLT3-L deprivation, the anti-proliferative effect observed upon NF-kB inhibition mostly relies on a limited number of genes directly regulated by MLL-AF9.

## Discussion

Over the last years, a large body of reports has highlighted the important role of epigenetic regulators in the pathogenesis of various types of cancer, including leukemias. One example of the latter is represented by MLL-fusion leukemias that, although characterized by an adverse prognosis, possess a relatively simple genetic background where the MLL-fusion gene is in most cases the only driver mutation [38]. Therefore, MLL-rearranged leukemias have been studied extensively to evaluate the influence of epigenetic regulators on the oncogenic transcription programs.

Wild-type MLL proteins contain a SET domain that harbors H3K4 methyltransferase activity allowing transcription initiation by Polymerase II [17]. Within MLL fusion proteins this SET domain is lost together with its catalytic activity, but MLL fusion proteins gain the ability to methylate H3K79 via recruitment of the H3K79 methyltransferase DOT1L [18,39]. As a consequence, the epigenetic landscape around promoters is changed resulting in transcriptome alterations with important consequences for leukemic transformation [18,39]. It is therefore no surprise that DOT1L inhibitors are currently extensively evaluated for their efficacy in MLL-rearranged leukemias [40–43].

In the present work, we aimed to identify targetable signaling networks in human MLL-AF9 leukemias. We show that MLL-AF9 cells critically depend on FLT3 ligand- induced pathways for their survival. Both the initiation of transformation as well as the maintenance of transformed leukemic cells critically requires the presence of FLT3-ligand. We also find that the FLT3-receptor is strongly upregulated by MLL-AF9, coinciding with a direct binding of MLL-AF9 to the FLT3 locus, thereby facilitating FLT3 signaling. Murine MLL-ENL-transformed cells have been shown to depend on FLT3-ligand, while MLL-AF9-induced transformation of murine cells did not depend on FLT3-ligand [44], in contrast to our findings. Possibly, differences in the role of FLT3 signaling in the murine and human hematopoietic compartments might play a role. The FLT3 receptor is for instance expressed in the most primitive human long-term HSCs, but not in murine LT-HSCs [45–47], and it is possible the FLT3 signaling is less important in the GMP-driven MLL-AF9 leukemia models in the mouse compared to more HSC-driven MLL-AF9 leukemia in human CB model systems [22]. In line with our findings, FLT3-L dependency has also been shown in human CB MLL-AF9 models by the Mulloy lab [48].

Downstream, we find that CB MLL-AF9 cells heavily depend on the activity of the serine/threonine kinase TAK1 and NF-kB. Previously, we and others identified that AML cells frequently display constitutive NF-kB activity [37,49,50] and heavily depend on TAK1 for their survival [35], and here we show that FLT3-ligand stimulated MLL-AF9 cells also do not tolerate inhibition of either TAK1 of NF-kB activity. An important role for NF-kB for MLL oncoproteins was also shown earlier [33]. Transcriptome studies revealed that deprivation of FLT3 ligand resulted in loss of genes that enriched for the GO terms DNA replication and G1/S cell cycle transition, coinciding with a loss of S/G2/M and accumulation of cells in G1 in cell cycle analysis studies, and also expression of the survival gene BCL2 depended on the presence of FLT3 ligand.

These data show strong overlap with a dependency of MLL-AF9 cells on BRD3/4 activity. We evaluated the *in vitro* and *in vivo* efficacy of the BRD3/4 inhibitor I-BET151 in various human MLL-AF9 (primary) models and patient samples and in line with what was published previously [8,51] we find good efficacy upon treatment with I-BET151. MLL-AF9 can transform CB CD34^+^ cell along the myeloid as well as the lymphoid lineage, and we find that both are strongly dependent on the presence of BRD3/4 activity. Interestingly, many of the genes regulated by BRD3/4 were not directly bound by MLL-AF9 as determined by ChIP-seq experiments. These data suggest that BRD3/4 does not directly control MLL-AF9 chromatin recruitment but rather suggest that it controls independent, but critically important, pathways for the survival of MLL-AF9 cells. While it was initially proposed that BRD4 would directly recruit MLL-fusion proteins to the chromatin it has now become clear that MLL-fusion proteins/DOT1L and BRD4 act independently, whereby DOT1L results in methylation of H3K79 followed by transcription factor-mediated recruitment of EP300 that deposits an acetyl mark on H4K5 [52]. This in turn forms a docking site for BRD4 thereby facilitating the target gene expression and co-inhibition of DOT1L and BRD4 has been shown to act synergistically in targeting MLL-rearranged leukemias [52].

Another interesting finding is that, although MLL-AF9 cells were sensitive to I-BET151, the sensitivity under liquid culture conditions was much stronger compared to the sensitivity observed under stromal co-culture conditions. Although the exact underlying mechanisms are not yet clear, it is quite possible that the bone marrow niche can provide protective signals for leukemic cells. For instance, survival pathways controlling BCL2 can also be activated by various (secreted) factors arising from stromal cells, a phenomenon that will certainly be further investigated in our future studies. Possibly, this also explains why we do not observe a complete eradication of MLL-AF9 cells in our in vivo humanized niche xenograft model in which leukemias are grown in the presence of a microenvironment composed of human mesenchymal stromal cells (Fig 1 and [32]). While we performed all the in vitro experiments on murine MS5 stroma supplemented with human cytokines in order to have a reproducible standardized system, it would certainly be of interest to evaluate drug dependencies in vitro in the presence of primary human MSCs as well.

In conclusion, a concept emerging from all our experiments is that although MLL-AF9 drives an aberrant gene expression program regulating for instance the HOXA cluster, it still critically relies on non-mutated transcription factors or tyrosine kinases. We show that BRD3/4 and the FLT3-TAK1/NF-kB pathways collectively control a set of targets that are critically important for the survival of human MLL-AF9 cells.

## Supporting information

S1 Table Transcriptome and ChIPseq data. (XLSX)

S1 Fig I-BET151 inhibits MLL-fusion leukemic cord blood models. Growth curves for CB MLL-AF9 MS5 co-cultures (myeloid (**A**) and lymphoid (**B**) conditions) upon I-BET151 treatment.
(PDF)

S2 Fig I-Bet151 targets specific and non-specific MLL-AF9 targets. **A**) Venn diagram for MLL-AF9 specific binding sites in THP1 cells [31] versus 3-fold upregulated genes upon 2 μM I-BET151 treatment in THP1 cells in a dose-ependent manner. **B**) ChIP-seq occupancy profiles of AF9, MLL, H3K79me2, H3K4me, H3K27Ac, H3K27me3 at the various loci. Examples of I-BET151 upregulated genes are shown without clear MLL-AF9 chromatin binding. RNAseq in THP1 cells was also performed and tracks are included as well.
(PDF)
