# Supplementary material for: BRD3/4 inhibition and FLT3-ligand deprivation target pathways that are essential for the survival of human MLL-AF9+ leukemic cells
Source: PLoS One. 2017 Dec 14;12(12):e0189102. doi: 10.1371/journal.pone.0189102 (PMC5730124; doi:10.1371/journal.pone.0189102)
Supplement: S1 Fig — Growth curves for CB MLL-AF9 MS5 co-cultures (myeloid (A) and lymphoid (B) conditions) upon I-BET151 treatment. (PDF) [file pone.0189102.s002.pdf]

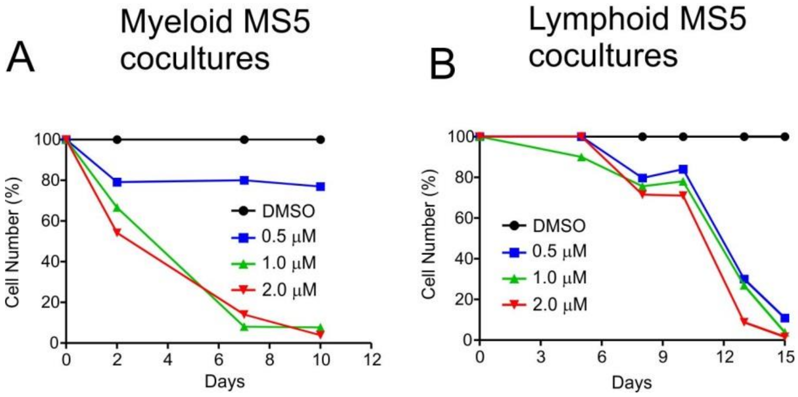

S1 Fig. I-BET151 inhibits MLL-fusion leukemic cord blood models. Growth curves for CB MLL-AF9 MS5 co-cultures (myeloid (**A**) and lymphoid (**B**) conditions) upon I-BET151 treatment.
