# Supplementary material for: BRD3/4 inhibition and FLT3-ligand deprivation target pathways that are essential for the survival of human MLL-AF9+ leukemic cells
Source: PLoS One. 2017 Dec 14;12(12):e0189102. doi: 10.1371/journal.pone.0189102 (PMC5730124; doi:10.1371/journal.pone.0189102)
Supplement: S2 Fig — A) Venn diagram for MLL-AF9 specific binding sites in THP1 cells [31] versus 3-fold upregulated genes upon 2 μM I-BET151 treatment in THP1 cells in a dose-ependent manner. B) ChIP-seq occupancy profiles of AF9, MLL, H3K79me2, H3K4me, H3K27Ac, H3K27me3 at the various loci. Examples of I-BET151 upregulated genes are shown without clear MLL-AF9 chromatin binding. RNAseq in THP1 cells was also performed and tracks are included as well. (PDF) [file pone.0189102.s003.pdf]

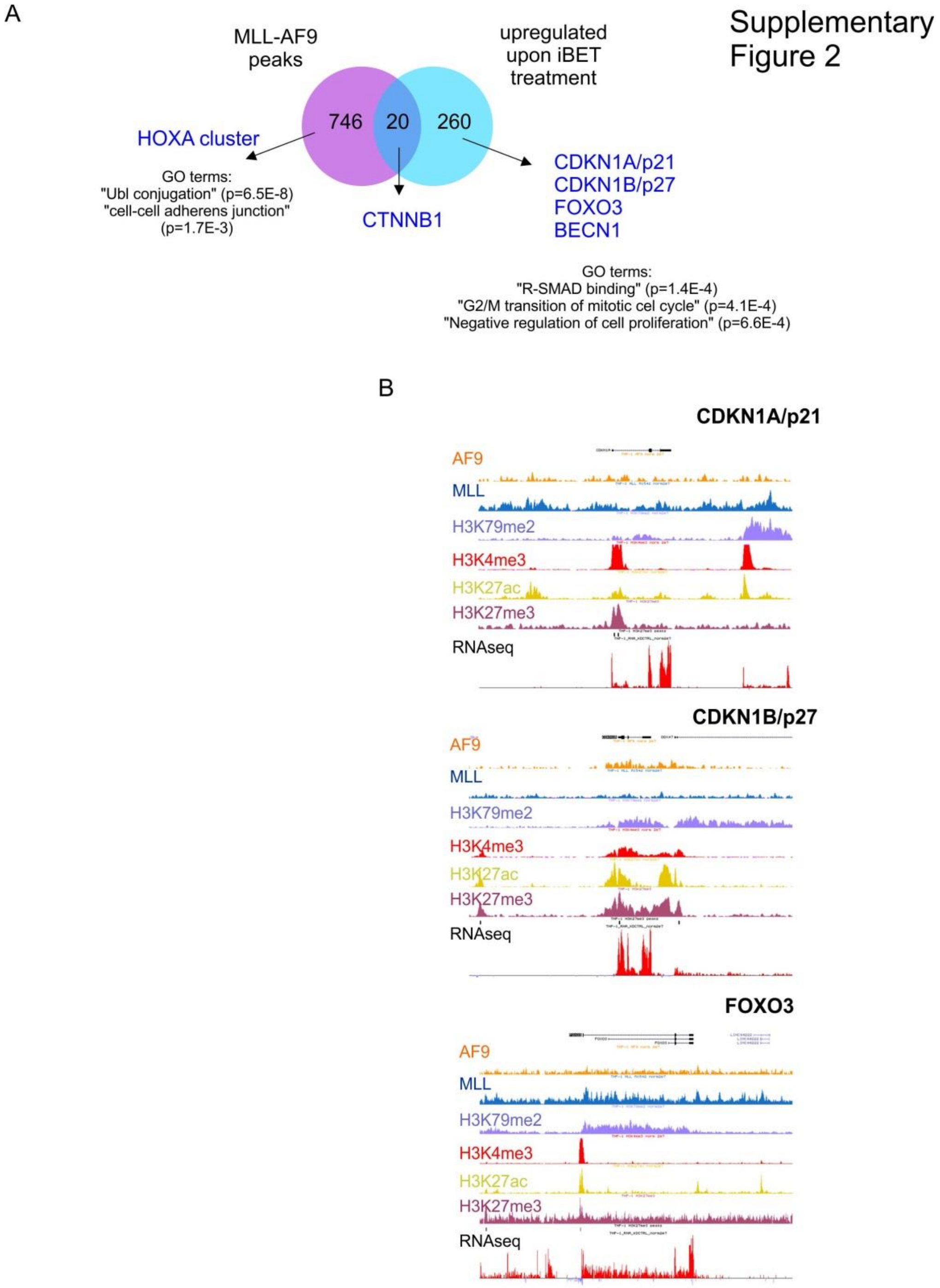

S2 Fig. I-Bet151 targets specific and non-specific MLL-AF9 targets. A) Venn diagram for MLL-AF9 specific binding sites in THP1 cells [37] versus 3-fold upregulated genes upon 2 μM I-BET151 treatment in THP1 cells in a dose-ependent manner. **B**) ChIP-seq occupancy profiles of AF9, MLL, H3K79me2, H3K4me, H3K27Ac, H3K27me3

at the various loci. Examples of I-BET151 upregulated genes are shown without clear MLL-AF9 chromatin binding. RNAseq in THP1 cells was also performed and tracks are included as well.
